# Cervical spondylotic myelopathy with vitamin B_12_ deficiency: Two case reports

**DOI:** 10.3892/etm.2013.1240

**Published:** 2013-07-30

**Authors:** YAO XU, WENJUN CHEN, JIANYUAN JIANG

**Affiliations:** Department of Orthopedics, Huashan Affiliated Hospital of Fudan University, Jing’an, Shanghai 200040, P.R. China

**Keywords:** vitamin B_12_, cervical spondylotic myelopathy

## Abstract

Although it has been observed that a vitamin B_12_ (VB_12_) deficiency may lead to defects in the nervous system, there is a lack of studies elucidating whether VB_12_ has a role in the pathogenesis of cervical spondylotic myelopathy (CSM). The present study describes two cases of CSM observed in the clinic, where the patients presented with common characteristics of the typical clinical symptoms; however, T2-weighted magnetic resonance imaging examinations revealed that although the degree of spinal cord compression was not serious, the spinal cord exhibited significant high signal changes. At the same time, the serum VB_12_ levels of the two patients were lower compared with those of normal controls. The symptoms of the patients improved following anterior cervical decompression surgery and VB_12_ replacement therapy. The incidence of CSM in the two patients may have been correlated with a lack of VB_12_. Therefore, it is recommended that the serum VB_12_ levels are checked in cases of CSM where the standard imaging and clinical manifestations do not fully match.

## Introduction

Cervical spondylotic myelopathy (CSM) is a cervical degenerative disease with a prognosis that is correlated with the cervical disc degeneration, the degree of spinal cord compression and the age of the patient (1,2). The prognosis of the disease is not optimistic and the occurrence of a gradual deterioration with time is high. To date, the pathogenesis of CSM has not been fully elucidated, although the major compression theory suggests that the symptoms of CSM are associated with the stenosis of the spinal canal and the degree of spinal cord compression (3). However, the extent of the compression does not fully explain the clinical symptoms of the spinal cord injury.

VB_12_ deficiency has been observed to lead to the development of degenerative diseases of the nervous system, including the peripheral and central nervous systems, and there have been numerous studies on subacute combined degeneration of the spinal cord resulting from VB_12_ deficiency (4). However, to date, there have been no studies concerning a VB_12_ deficiency occurring in combination with CSM, to the best of our knowledge. This case report describes two cases of CSM observed in the clinic, where the patients also presented with VB_12_ deficiency. This study was approved by the ethics committee of Huashan Affiliated Hospital of Fudan University (Shanghai, China). Informed consent was obtained from all patients.

## Case report

### Case one

**Figure 1.**
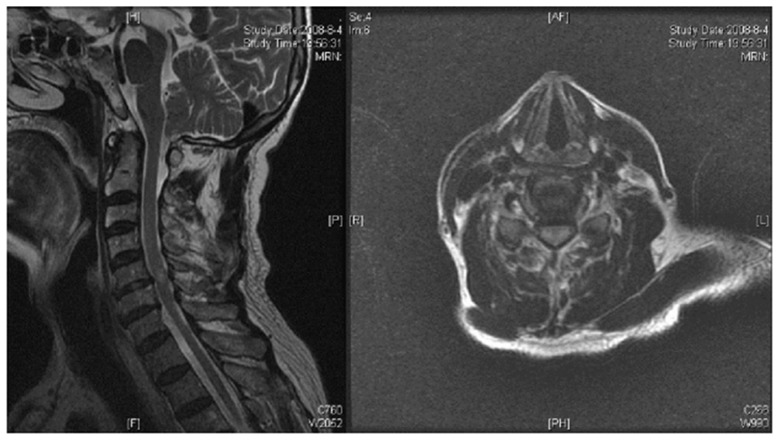
Case 1. Sixty-eight-year-old male exhibiting disc herniation at the C5–6 level. T2-weighted magnetic resonance imaging (MRI) revealed high signal changes in the spinal cord.

A 68-year-old male was admitted to the clinic. The patient had a history of a fall two weeks prior to the admission to the clinic, although no direct external force had been applied to the head and neck during the fall. The patient complained of numbness in the forearms and hands, tingling sensations and a lack of power when clenching the fists. Through the physical examination, it was observed that the flexion and extension range of the patient’s body and neck were normal and that there was no sign of tenderness. The flexion and extension strength of the patient’s elbow muscle was of Grade III, there was numbness and hyperalgesia in the bilateral forearm and hand, the grip of the patient’s two hands was weak, the bilateral Hoffmann sign was (+) and the lower extremity muscle strength was graded as Class IV. Furthermore, the muscle tone was normal, the tendon reflex was (+++), the patellar clonus was (−) and the ankle clonus was (−). Cervical magnetic resonance imaging (MRI) examination revealed that cervical disc degeneration was apparent at C5–6 level with backward extrusion, and that there were high signal changes within the same level of the spinal cord. Mild dural compression was observed in the cross section (Fig. 1). According to the study by Nagata *et al* (5), the degree of spinal cord compression may be classified into one of four groups: Level 0, no pressure on the spinal cord; Level 1, mild compression on the spinal cord; Level 2, the degree of spinal cord compression is <1/3; Level 3, the degree of spinal cord compression is >1/3.

In this case, the degree of spinal cord compression on the MRI imaging was classed as Level 1, i.e. only mild compression; however, T2-weighted (T2W) MRI examination revealed that there were high signal changes in the spinal cord. Therefore, although the clinical manifestations and symptoms indicated damage to the central beam of the spinal cord, this was not consistent with the mild degree of compression. Laboratory examinations revealed that the level of serum VB_12_ was 173 pg/l, which was below the normal range (211–946 pg/l). It is possible that VB_12_ deficiency may be the cause of spinal cord injury in this patient in whom there is only a mild degree of spinal cord compression.

**Figure 2.**
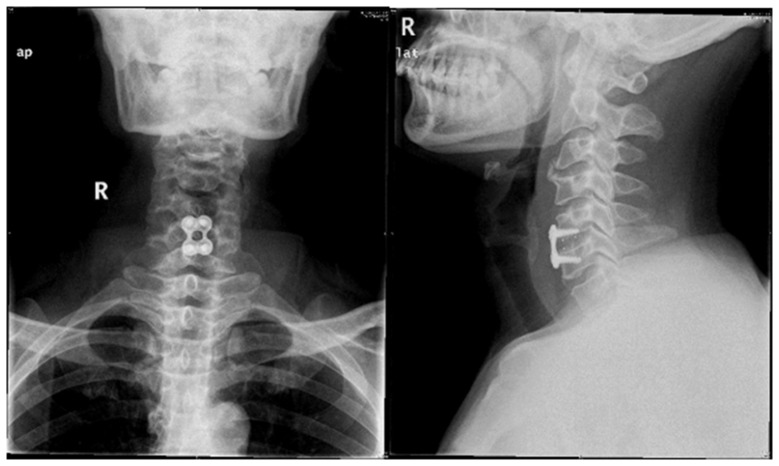
Case 1. X-ray examination three months subsequent to the C5–6 anterior decompression-fusion and internal fixation surgery.

The patient underwent anterior cervical decompression-fusion and internal fixation (Fig. 2), at the same time as receiving three months of oral VB_12_ replacement therapy. One week later, the tingling feeling in the patient’s hands was significantly improved and his fist-clenching ability was restored and two weeks later the patient’s muscle strength reached Grade III; three months subsequently, the numb feeling in the forearms and hands of the patient had almost disappeared, the patient’s finger activities had returned to normal and the fist muscle strength had recovered to Class IV.

### Case two

**Figure 3.**
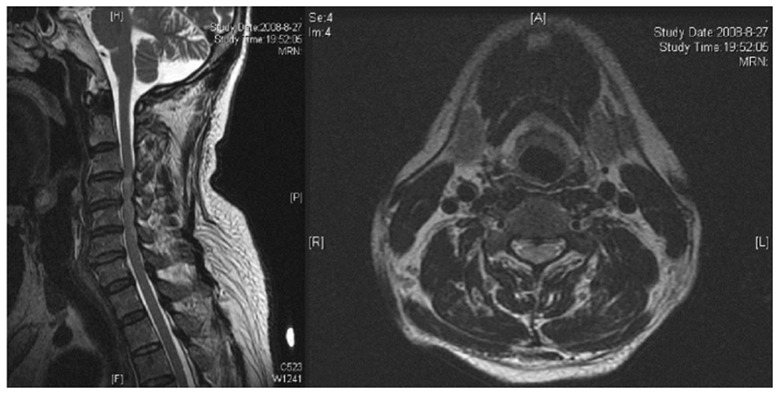
Sixty-one-year-old male exhibiting high signal spinal cord changes at the C3–4 level with T2-weighted magnetic resonance imaging (MRI).

A 61-year-old male was admitted to the clinic with numb upper limbs, feelings of weakness and an unstable gait that had been apparent for more than one month. The patient complained of a sense of strapping in his chest and an inability to grip with his hands or hold objects without dropping them. Furthermore, the patient had weak fine finger movements, such as writing, and felt his walking was like walking on cotton. Physical examination revealed normal neck and bilateral shrug activities, Grade IV flexion and extension strength of the elbow muscle, numbness in the bilateral forearms and hands and weak fist-clenching abilities. The bilateral Hoffmann sign was (+), the lower extremity muscle strength was of Grade IV, the muscle tension was high and the tendon hyperreflexia was (++++). The cervical MRI revealed high signal changes at the C3–4 level in the spinal cord and slipped discs were observed at C3–4 and C4–5 (Fig. 3).

The MRI examination indicated that the compression at the C3–4 level of the patient’s spinal cord was of Level 2, according to the Nagata grading, and T2W MRI revealed high signal changes within the same level of the spinal cord. In addition, the patient exhibited symptoms typical of motor neuron injury in CSM. Laboratory tests measured that the level of serum VB_12_ was 182 pg/l, which was below the normal range (211–946 pg/l). Although the spinal cord compression was not serious, there were high signal changes in the intramedullary spinal cord that were apparent with T2W MRI, and the clinical manifestations were typical of spinal cord injury. The VB_12_ deficiency of the patient may have been the factor leading to the spinal cord damage.

Similar to the patient in case one, the patient in the present case underwent anterior decompression-fusion and internal fixation and, following three months of VB_12_ replacement therapy, the balance of the patient when walking improved markedly and the sense of strapping in the chest disappeared, although the numbness of upper limbs was still apparent. Furthermore, the fist action of the patient recovered and muscle strength was measured to be Grade III.

## Discussion

CSM is one of the main types of cervical degenerative disease, with clinical manifestations that are indicative of spinal cord compression. The development of CSM is normally slow and insidious, making early diagnosis and treatment a challenge. By the time it is posssible for a clear clinical diagnosis to be made, the condition of the patients has often deteriorated beyond the point when treatment is most efficacious (6).

The pathogenesis of CSM is not fully understood, although its incidence is correlated with the volume of the spinal canal, the degree of disc degeneration and spinal cord compression (7). Mechanical compression is considered the primary factor leading to the functional injury of the spinal cord; however, the compression theory alone is not able to explain all the clinical manifestations. It has been suggested that the apolipoprotein E allele increases the chances of CSM developing in patients under long-term spinal cord compression (8). Histological examinations of the pathological changes in patients with CSM have demonstrated mildly atrophic white matter, reductions in the number of neurons and pale medullary sheaths in the lateral and posterior cord (9).

It has often been observed in the clinic that certain CSM patients with a severe slipped disc and spinal cord compression on imaging do not have a spinal cord injury in the area of the clinical manifestations, while others exhibit a mild slipped disc and mild spinal cord compression on imaging, yet have a severe spinal cord injury in the area of the clinical manifestations. The degree of spinal cord compression on imaging and the clinical spinal cord injury performance are, therefore, not always consistent. This led to the consideration of whether there were other factors affecting the ability of the spinal cord to bear mechanical pressure.

VB_12_ is an indispensable catalyst of nucleic acid synthesis and protein metabolism in cells *in vivo*; at present, VB_12_ deficiency is a common disease (10,11). VB_12_ deficiency leads to a wide range of neuropathies, of which subacute combined degeneration of the spinal cord is particularly common. Neural changes caused by VB_12_ deficiency may be correlated with subcortical dysfunction and neuronal metabolism changes (12). Dalla Torre *et al* described two cases of isolated sensory axon neuropathy resulting from VB_12_ deficiency, and suggested that there was a requirement for serum VB_12_ levels to be detected for distal symmetric neuropathy (13). There have been rare studies on the role of VB_12_ in cervical spinal cord diseases and it is used as a conventional neurotrophic drug; however, there have been no studies concerning the effects of VB_12_ on the pathogenesis of spinal cord disease.

The two cases described in this study had the same characteristics, such as a shorter duration of time before occurrence of typical symptoms, and the clinical manifestations of typical CSM symptoms, including unsteady gait, a sense of strapping in the chest, numb hands, reduced fist muscle strength and pathological signs, such as the Hoffmann sign (+). The patients were not receiving oral VB_12_ treatment. MRI examinations revealed that the degree of spinal cord compression was not severe at Levels 1–2, although high signal changes on T2W MRI were observed within the spinal cord. Laboratory tests revealed that the serum VB_12_ levels were below the normal range, classing the patients as having a VB_12_ deficiency, which may have been one of the factors leading to the cervical spinal cord disease. Following anterior cervical decompression-fusion and internal fixation and VB_12_ replacement therapy, the clinical symptoms of the two patients significantly improved.

At present, there is still a lack of investigation into the role of VB_12_ in the pathogenesis of CSM. The two cases in the present study indicated that the detection of the VB_12_ levels of patients who do not exhibit serious spinal cord compression on imaging may be helpful in explaining the disease. Further studies to investigate the incidence of VB_12_ deficiency in patients with cervical myelopathy may be of value.

In conclusion, the two cases described in the present study suggested that the incidence of CSM may be correlated with VB_12_ deficiency, particularly for cases in which the clinical manifestations and the imaging do not fully match. In cases such as these, it may be beneficial to check serum VB_12_ levels.
